# The role of the insula in intuitive expert bug detection in computer code: an fMRI study

**DOI:** 10.1007/s11682-018-9885-1

**Published:** 2018-05-09

**Authors:** Joao Castelhano, Isabel C. Duarte, Carlos Ferreira, Joao Duraes, Henrique Madeira, Miguel Castelo-Branco

**Affiliations:** 1 ICNAS/CIBIT, Coimbra Institute for Biomedical Imaging and Life Sciences, University of Coimbra, Azinhaga de Santa Comba, 3000-548 Coimbra, Portugal; 2 CNC.IBILI, Faculty of Medicine, University of Coimbra, 3000-548 Coimbra, Portugal; 3 CISUC-DEIS, Polytechnic Institute of Coimbra, Coimbra, Portugal; 4 CISUC-DEI, University of Coimbra, Coimbra, Portugal

**Keywords:** fMRI, Brain mapping, Insula, Informatics, Error monitoring, Salience network, Computer code understanding

## Abstract

Software programming is a complex and relatively recent human activity, involving the integration of mathematical, recursive thinking and language processing. The neural correlates of this recent human activity are still poorly understood. Error monitoring during this type of task, requiring the integration of language, logical symbol manipulation and other mathematical skills, is particularly challenging. We therefore aimed to investigate the neural correlates of decision-making during source code understanding and mental manipulation in professional participants with high expertise. The present fMRI study directly addressed error monitoring during source code comprehension, expert bug detection and decision-making. We used C code, which triggers the same sort of processing irrespective of the native language of the programmer. We discovered a distinct role for the insula in bug monitoring and detection and a novel connectivity pattern that goes beyond the expected activation pattern evoked by source code understanding in semantic language and mathematical processing regions. Importantly, insula activity levels were critically related to the quality of error detection, involving intuition, as signalled by reported initial bug suspicion, prior to final decision and bug detection. Activity in this salience network (SN) region evoked by bug suspicion was predictive of bug detection precision, suggesting that it encodes the quality of the behavioral evidence. Connectivity analysis provided evidence for top-down circuit “reutilization” stemming from anterior cingulate cortex (BA32), a core region in the SN that evolved for complex error monitoring such as required for this type of recent human activity. Cingulate (BA32) and anterolateral (BA10) frontal regions causally modulated decision processes in the insula, which in turn was related to activity of math processing regions in early parietal cortex. In other words, earlier brain regions used during evolution for other functions seem to be reutilized in a top-down manner for a new complex function, in an analogous manner as described for other cultural creations such as reading and literacy.

**Electronic supplementary material:**

The online version of this article (10.1007/s11682-018-9885-1) contains supplementary material, which is available to authorized users.

## Introduction

Software programming is a complex and phylogenetically very recent human activity, even more than reading and literacy. In neuroscientific terms, it requires the expert integration of mathematical, including logical thinking and symbol manipulation, and language skills at an abstract level. Programming languages are universal in the sense that they do not depend on the native language of the programmer, which further enhances the interest to study this type of complex expertise from the neuroscientific point of view.

From a practical point of view this human ability is at the basis of one of the biggest industry sectors in the world: the software development industry, where error monitoring is critical. The global annual cost of information technology (IT) failures worldwide reaches stunning figures, ranging from $3 to $6.2 trillions, (CompTIA 2016). A large share of IT failures results from software faults (usually known as bugs), which are in fact the consequence of human errors in the software development process (Christmansson and Chillarege 1996; Duraes and Madeira 2006; Natella et al. 2013). It is therefore relevant to understand the neural basis of such errors, which might also generalize to other complex skills. Despite decades of intensive research on software engineering, a breakthrough in software reliability improvement has not been reached yet. Even when software is developed using highly mature development processes, the deployed software still has a relatively high density of residual bugs (Boehm et al. 2002; Duraes and Madeira 2006; Honda and Yamada 2012). With the huge dependency of our society on IT and software, bad quality software caused by residual bugs represent one of the most enduring and difficult technical challenges.

Despite decades of SW reliability research, software bugs have never been investigated from a neuroscience perspective. We aimed to find the neural underpinnings associated to human error in such highly abstract and complex intellectual task such as software programming. Furthermore, any possible insight on the deep reasons why human fails so often in software programming and debugging may have a big impact not only on software quality improvement, but also on other areas of human behavior requiring complex skills.

A previous proceedings report of Siegmund et al. (2014) suggested that software programmers strongly recruit language regions of the brain to understand source code. However, Siegmund et al. (2014) did not consider specific cognitive processes involving software related error monitoring and decision-making, nor did the analyzed programs contain bugs. Their task was focused on reading and understanding source code, which could include syntactic errors (but no real bugs), which likely increase the likelihood of activating areas related to conventional language processing (Siegmund et al. 2012). It is worth noting that software syntactic errors are always detected by compilers (i.e., they are not a technical problem for the software industry), while real bugs are related to the program semantics and are very hard to find.

Cultural creations of neuroevolutionary relevance, such as reading or mathematical thinking, have been proposed to reutilize cortical circuits that have evolved for different purposes (Dehaene 2013). Dehaene et al., have proposed that cortical regions maybe partly recycled for new human-specific uses (Dehaene et al. 2015). In other words, a brain region that evolved for a given processing demand might be reutilized when new demands emerge during human history for a given new function (Cohen et al. 2000).

Regarding language related ‘errors’ a few fMRI studies showed an involvement of the expected frontal and temporo-parietal regions in semantic analysis of anomalous sentences (Kuperberg et al. 2000). Left inferior frontal gyrus and clusters in frontal Brodmann areas 44, 45 and 46 (S. D. Newman et al. 2012), superior frontal cortex and right middle temporal gyrus activations are related to processing of anomalous sentences, syntactic and semantic violations respectively (A. J. Newman et al. 2001). However, software analysis/error monitoring require complex cognitive operations that deal with abstract concepts and software program structures, which are far beyond common language processing.

Given the particular nature of software programming constructs and logical operations (e.g., conditionals and loops) and related calculations, sorting, recursivity and working memory load, we performed a whole brain fMRI study to investigate the role of regions (e.g. insula, ACC) within the salience network in this relatively recent skill requiring complex processing. We expected to find effective connectivity going beyond the usual separation of semantic/syntactic error related areas (BA 9, 21, 44, 47) (A. J. Newman et al. 2001) and areas involved in math processing (in the parietal and frontal lobes, BA 7, 40) (Zago et al. 2001). Moreover, we expected to elucidate the role of distinct prefrontal regions involved in causal reasoning (middle frontal BA46, BA 9, 10) and necessary for binding information from different mnemonic sources (Baddeley 2000; Baddeley and Hitch 1992) and calculation or language processing. The conference report of Siegmund et al., did not find activation in math processing related regions such as in the intraparietal sulcus. This possibly because error detection just involved the type of simple syntactic analysis required by language processing (Amalric and Dehaene 2016; Dehaene et al. 1999; Desco et al. 2011; Gruber et al. 2001; Maruyama et al. 2012).

We tried to clarify the neural correlates of error monitoring during deep source code analysis using fMRI in a task requiring expert decision-making and bug detection during a code-inspection task by programming specialists. We created conditions similar to software code inspections as used in industry, where a software inspector (i.e., a software specialist) analyses code excerpts to try to find possible bugs. In our experiments, faults (bugs) have been previously seeded in the programs, using well accepted fault models that represent both realistic and representative faults found in real software in the field (Duraes and Madeira 2006; Perry and Evangelist 1996; Sullivan and Chillarege 1991). Moreover, we aimed to identify neural patterns relating to code understanding with an emphasis on two particular moments: first, when the inspector suspects he has identified a bug (‘eureka moment’, but uncertainty still present), which is based on intuition; and second, the moment when the participant reports definite confidence on bug detection (confirmation/high certainty). We expected a delay of several seconds from intuition to formal confirmation, as confidence levels increase based on evidence accumulation.

Giving the nature of our task (participants have to decide whether a given portion of code contains a bug), we expected to identify a relevant contribution from regions within the salience network. This include regions such as the insula, and other expert decision-making related brain regions (Buckley et al. 2009; Hauke R Heekeren et al. 2008; Kennerley et al. 2011; Stoewer et al. 2010; Wallis 2007; Wunderlich et al. 2012). The lateral orbitofrontal cortex, the ventromedial prefrontal cortex and adjacent medial orbitofrontal cortex (OFC), anterior cingulate cortex (ACC), and the anterior lateral prefrontal cortex have been related to reward-guided decision-making both in humans and animal models (Neubert et al. 2014, 2015; Rushworth et al. 2011; Sallet et al. 2013). The activation of these regions was also expected from the inherent reward value of the eureka detection moment. Previous studies showed that OFC integrates multiple sources of information to reach a motivated decision (Wallis 2007). The information integrated and processed in medial prefrontal cortex (Wunderlich et al. 2012) can then be held in working memory where it can be used by lateral prefrontal cortex to plan and organize actions (Wallis 2007) towards source code debugging. Moreover, the ACC is a region of the salience network which is critical in encoding choice predictions and prediction errors using a common valuation currency reflecting the integration of multiple decision parameters (Kennerley et al. 2011; Wunderlich et al. 2012). However, brain regions playing a key role during decision processes and error monitoring (Iannaccone et al. 2015) include not only the anterior cingulate, prefrontal cortex (Domenech and Koechlin 2015; Krawczyk 2002), but also the insula (Bastin et al. 2017; Castelhano et al. 2014; Droutman et al. 2015) which is modulated by task difficulty and uncertainty levels (Lamichhane et al. 2016). We thus asked whether activity in these brain regions within the salience network is predictive of bug detection accuracy during source code debugging. These results can make an important contribution to our understanding of the neural mechanisms related to error monitoring during tasks requiring complex integration of mathematical and general logical and language skills such as software programming.

## Material and methods

### Participants

We recruited a group of 20 software-development professionals. All of them had formal master and/or PhD degrees in informatics or equivalent and more than 3 years of experience with C language programming. All had previously realized code-inspection during their career and had strong experience as formal code inspectors. One was excluded due to having exceeded the motion criteria inside the scanner, rendering the scanning data unusable. The participants included in the analysis have a mean age of 28 years. 18 out of 19 participants are male and right handed, but the whole group used the joystick (to control the task) with the right hand due to individual personal preference. All the participants had normal or corrected to normal vision. The study was approved by the Ethics Committee of the Faculty of Medicine of the University of Coimbra, in accordance with the Declaration of Helsinki and all experiments were performed in accordance with relevant guidelines and regulations. Informed consent was obtained from all participants. The data associated with this research are available upon request to the corresponding author.

### Source code description

We used source code blocks, written in C language as a main stream programming language, with 20 to 60 lines of code (see examples in Figs. A.1 to A.3) so that the subject would be able to identify faults in the allotted time, using a joystick for line navigation and button choice selection (see video A.1 highlighting task details). For details about these source codes please refer to appendix A. As software inspections are used in relatively small program segments (to limit the duration of the inspection), we selected some examples of representative programs with adequate size for inspection in a single session. The programs used are C implementations of Quick sort, Shell sort, and Matrix multiplication. The programs have been previously seeded with realistic bugs (Quick sort with 7 bugs, Shell sort with 4 bugs and Matrix multiplication with 4 bugs) and are inspected (in the fMRI session) at a random order. There are also three control programs with no faults (neutral code) that are used by inspectors to perform a simple mental reading of the neutral code to understand what the instructions do. Although the main goal is to contrast between bug suspicion and detection we also aimed to investigate the contrast between code reading/understanding and the search for a bug. These programs (neutral code vs. code with bugs) have a relatively similar complexity as calculated by the Modified cyclomatic McCabe algorithm (average complexity of neutral codes: 3.33 ± 2.65; average complexity of code with bugs: 3.0 ± 2.27; U = 21, *p* = 0.755).

**Fig. 1:**
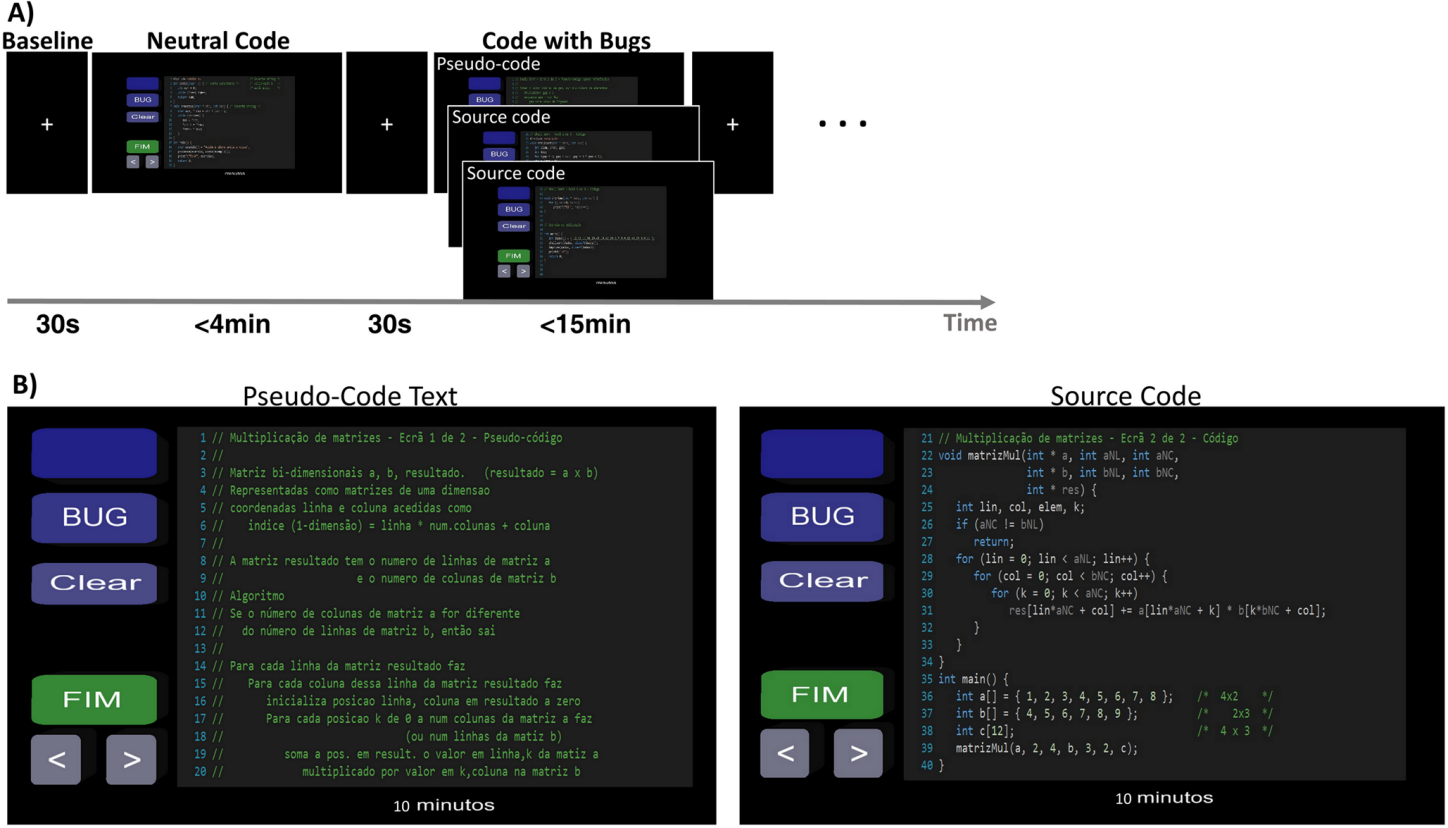
Example of task timeline of one run. **a** Subjects were presented with the code in the centre of the screen and navigated through the code with an fMRI compatible joystick. 6 types of programs (3 neutral code without bugs and 3 codes with bugs) were presented divided by two runs. The codes were separated by a baseline block and the order of presentation was randomized. Following the logic of the code inspections as performed in the industry, subjects are given the tools to help find bugs, including the explanation of what the code is about (except the code itself) outside the MRI scan (including a training session). **b** Screen snapshot. Left: example of pseudo-code screen (text trial). Right: example of source code with bugs. Subjects were instructed to detect bugs and press the line with the bug or suspicion as soon as possible. The bug had to be confirmed in the BUG button available in the left pannel. Subjects were free to explore and change to the pseudo-code and source code screens as many times as needed (‘< >’ buttons). Each block of code for inspection has a maximum duration but ends when the subject press the ‘FIM’ (meaning “END”) button (this happens when the subject is confident that he/she has found all the bugs)

We design the task in such a way as to be as close as possible to real-life source code inspection, to try to increase the ecological validity of the study while ensuring a sufficient number of trials to allow for adequate statistical power. Programs were shown to participants as a series of screens containing 20 lines of source code at a time. The first screen/page of the programs with faults was a text description of the algorithm in pseudo-code to help the subjects recall the logic and goal of the code shown (Fig. 1, and Video A.1). Subjects were free to explore and navigate to the pseudo-code and source code screens as many times as needed. Besides the code, the screen also contains control buttons to navigate in the code and to record/unregister a given line as containing a fault. Before the fMRI session, the participants received a detailed explanation of the programs goals, structure, and algorithm. Subjects performed a brief training session outside the scanner to make sure that they were familiarized with the task procedures inside the scanner. In order to reinforce the debugging process, all programs include a *main* function to help contextualize their use, namely by showing an example of inputs and how the results could be displayed. Participants were told that faults could exist anywhere in the code, including in the *main* function.

### Task details

Stimulation was implemented with virtual-reality toolkit Vizard (WorldViz, Santa Barbara, CA). The stimuli were presented in an LCD monitor (NordicNeuroLab, Bergen, Norway) placed approximately 156 cm away from the participants’ head (that could be seen through a mirror system mounted above the participant’s head), with a frame rate of 60 Hz and dimensions of 698.40 × 392.85 mm. All subjects were shown the above-described programs in a random order (three stimuli with bugs and three neutral code without bugs, plus eight baseline blocks) divided into two runs to avoid subject fatigue. Subjects were told which blocks included errors and which were error free. The participants were instructed to understand and identify bugs in the source code and signal the events in the corresponding button of the screen (Fig. 1). We used the neutral code as a secondary control condition without bugs, although the main contrast of interest was bug suspicion vs. bug detection. In the latter case, subjects were asked to follow the code lines with the joystick, as a pointer while reading/understanding the code, to match for movement patterns. Participants were able to activate the controls and navigate through the code using an fMRI compatible joystick (Hybridmojo, San Mateo CA, USA). Participants were also instructed to select a line as soon as they suspected the presence of a bug (‘suspicion/eureka moment’) and to confirm the bug in the button ‘Bug’ when they were sure that there was a fault (‘Bug detection’). The timings of these actions (behavioural data) were synchronous with fMRI data and used as predictors for functional data analysis. The same source codes were used for all subjects but the order of the blocks and runs were randomized and counterbalanced between subjects to avoid order effects (see Fig. 1 and video A.1 for an example of the task). The duration allowed for each condition was as follows: baseline 30s; neutral code <4 min; code with bugs (Quick sort <15 min; Shell sort <15 min; Matrix Multiplication <10 min). See Table A.1 for a group summary of durations per stimulus and subject, as each experimental block ends when the subject is confident that he found all the bugs.

### Behavioral and eye tracking acquisition and analysis

Behavioral data (button presses) were simultaneously recorded during fMRI. Analysis was then performed offline. The stimulus duration, suspicion events and bug detection events (true positive, false positive and clear bugs) were extracted and precision (true positive / (true positive + false positive)) % measures were calculated per subject.

Eye tracking data were recorded to visually assess online the compliance of the subject with the task and to control for attentional allocation. An EyeLink 1000 (SR Research Ltd.) long-range mount, binocular (corneal reflection and pupil tracking) fMRI compatible eyetracker was used to record eye movements during the fMRI sessions with a sampling rate of 500 Hz. The default 9-point calibration of the eye tracking system was performed before the functional runs, including a validation step (recalibration was performed if necessary to achieve good tracking accuracy; two subjects did not yield eye tracking data of sufficient quality). Saccade events were generated online by the EyeLink eye tracker using a default internal heuristic saccade detector. Subsequent analyses were performed offline in Matlab R2013a (Mathworks, USA). An area-of-interest (AOI) analysis was implemented for the source code with bugs. We classified fixations into one of two categories: fixations inside bug AOIs, fixations outside bug AOIs. Fixation parameters (number of fixations, fixation duration min, max and mean values) were extracted per time-window of source code and the suspicion and bug detection events. We performed statistical t-tests (alpha 0.05) to assess the differences between fixation patterns inside vs. outside bug AOIs (IBM SPSS statistics v22).

### fMRI acquisition parameters

We acquired anatomical and functional data in a 3 T Magnetom Trio Tim MRI scanner (Siemens, Erlangen, Germany) with a 12-channel head coil. Anatomical images were acquired using MPRAGE sequence at an isotropic resolution of 1 mm^3^. This information was used for further co-registration with functional data. Regarding functional images, EPI sequences were acquired with slice thickness of 3 mm and voxel size 4 mm^2^, 36 slices covering the whole brain, with repetition time 3000 ms, echo time 30 ms, flip angle of 90°, matrix size 256 × 256 and field-of-view of 256 × 256. Participants underwent 2 functional runs each.

### fMRI analysis

fMRI analysis was performed using BrainVoyager QX 2.8 (BrainInovation). The preprocessing was performed with the default parameters (cubic spline slice scan time correction; trilinear 3D motion correction; GLM with Fourier basis set with 2 cycles high-pass filtering). Structural and functional data were coregistered and transformed to the standard Talairach space. A random effects General Linear Model (K J Friston et al. 1995) multi study/subject analysis was then performed to assess the brain activity patterns. Only the subjects with 5 bugs or more were included in the group analysis (*N* = 17). The baseline blocks were defined for the time-windows without a task (fixation cross). We used the eye tracker data to help set a semantic/reading predictor as defined by the period the participants were spending time reading an explanatory window of source code with bugs, the pseudo-code. Blocks of source code with bugs were divided in: Pseudo-code (blocks of text reading); Source code (blocks of source code screens with bugs). The number of trials and duration of each block are summarized in Table A.1.

The Bug detection predictor included true and false positive bugs. True and false suspicions predictors were also pooled together since no differences were found in the contrast between true positive and false positive suspicions or bug detection responses (nor in suspicion and bug detection events separated by small and large delays) in an exploratory analysis.

We defined the following contrasts regarding source code debugging:

1. As the main contrast, in order to study the functional differences in suspicion and bug detection during code-inspection, we defined a contrast during the search for a bug component of the paradigm: 1) Suspicion (the first moment a bug is signaled) vs. Bug detection (when a ‘BUG’ is confidently confirmed). We then computed a random effects group analysis. The average delay between suspicion and bug detection were 10 s (of evidence accumulation) thus the potential temporal correlation/overlap responses between them due to the hemodynamic delay is minimal.

As secondary contrasts we also investigated:

2) understanding software source code (contrast between source code blocks, with and without bugs, and pseudo-code/text periods); 3) searching for a real bug (source code with bugs vs. neutral code).

Data were corrected for multiple comparisons with the False Discovery Rate (FDR) approach at the single voxel level (*p* < 0.05). A cluster threshold was also applied to discard small clusters (<150 voxels). Note that correction with FDR at the single voxel level and cluster threshold instead of FWE makes a compromise between the limitations inherent to type II and type I errors. Furthermore, the beta values (Suspicion vs. Bug detection events) were extracted from the resulting region of interest (ROI) in regions of the SN and in particular the insula and a Pearson correlation analysis of these values and behavioral precision (% of correct bug detections) was computed.

### Connectivity analysis

We aimed to clarify the roles of different nodes within the saliency network, but with no additional a priori hypothesis. The insula has been reported as a key node of the saliency network, executive-functions and decision-making tasks (Sridharan et al. 2008). We thus calculated the Granger causality map (GCM plugin tool from Brainvoyager) to find the regions that influence/are influenced by the insular ROI. We computed GCM that is a method exploring directed influences (effective connectivity) between distinct regions in fMRI data without an a priori model of assumed regional connections between regions. This plugin computes GCM with respect to a single region-of-interest using temporal precedence information to compute Granger causality maps and identify voxels that are sources or targets of the selected ROI. Briefly, it makes use of Granger concept to determine if the past of a time-series may improve the prediction of the current value of another time-series (Roebroeck et al. 2005). In this way, one could map our ROI influence over the brain with a measure of effective connectivity. The instantaneous correlation/functional connectivity without a direction was also computed. The difference between influence (from the insula, because of its identified relation with precision of bug detection) and influence from the voxel to the reference region is finally extracted. The positive value represent voxels where influence from the reference region dominates (i.e. that are targets of the ROI) and the negative value at voxels signal the voxels where influence to the reference region dominates (i.e. that are sources of influence to the ROI).

## Results

### Behavioral and Eyetracking results

The programming experts reached a relatively high precision for bug detection/confirmation: group median true positives: 7 (min 5, max 8); false positives: 2 (min 1, max 8). The same bugs reported more than on one occasion were only considered once for behavioral analysis. The median number of true suspicion events was 9 (min 5, max 14) and false suspicion events was 3 (min 1, max 10).

The eyetracking data unraveled the expected patterns of fixations during source code debugging. We performed an area-of-interest (AOI) analysis defined for the code lines with bugs during source code inspection and the suspicion and bug detection moments. As trivially expected, the number of fixation in/out bug AOI differed significantly (*t* = −6.60, *p* < 0.00001) but this effect disappears if one normalizes the data for the size of the AOIs. Moreover, the mean fixation duration in/out bug AOI did not differ (*t* = −1.387 *p* = 0.177). Furthermore, the number of fixations (*t* = −0.678 *p* = 0.513) and mean fixation duration (*t* = 0.295 *p* = 0.774) of the Suspicion vs. Bug detection events did not differ. Supplementary Table A.2 summarizes the descriptive statistics and Fig. A.4 shows an example of the tracking scan path for one subject. Notably, we did not observe significant correlations between Suspicion and Bug detection fixation durations (*r* = 0.21, *p* = 0.53).

### The neural basis of software source code understanding

We investigated the neural underpinnings of software program processing in expert participants by using fMRI while subjects performed a bug detection task. The task included conditions with and without bugs, and the main contrast of interest was bug suspicion vs bug detection.

**Fig. 2:**
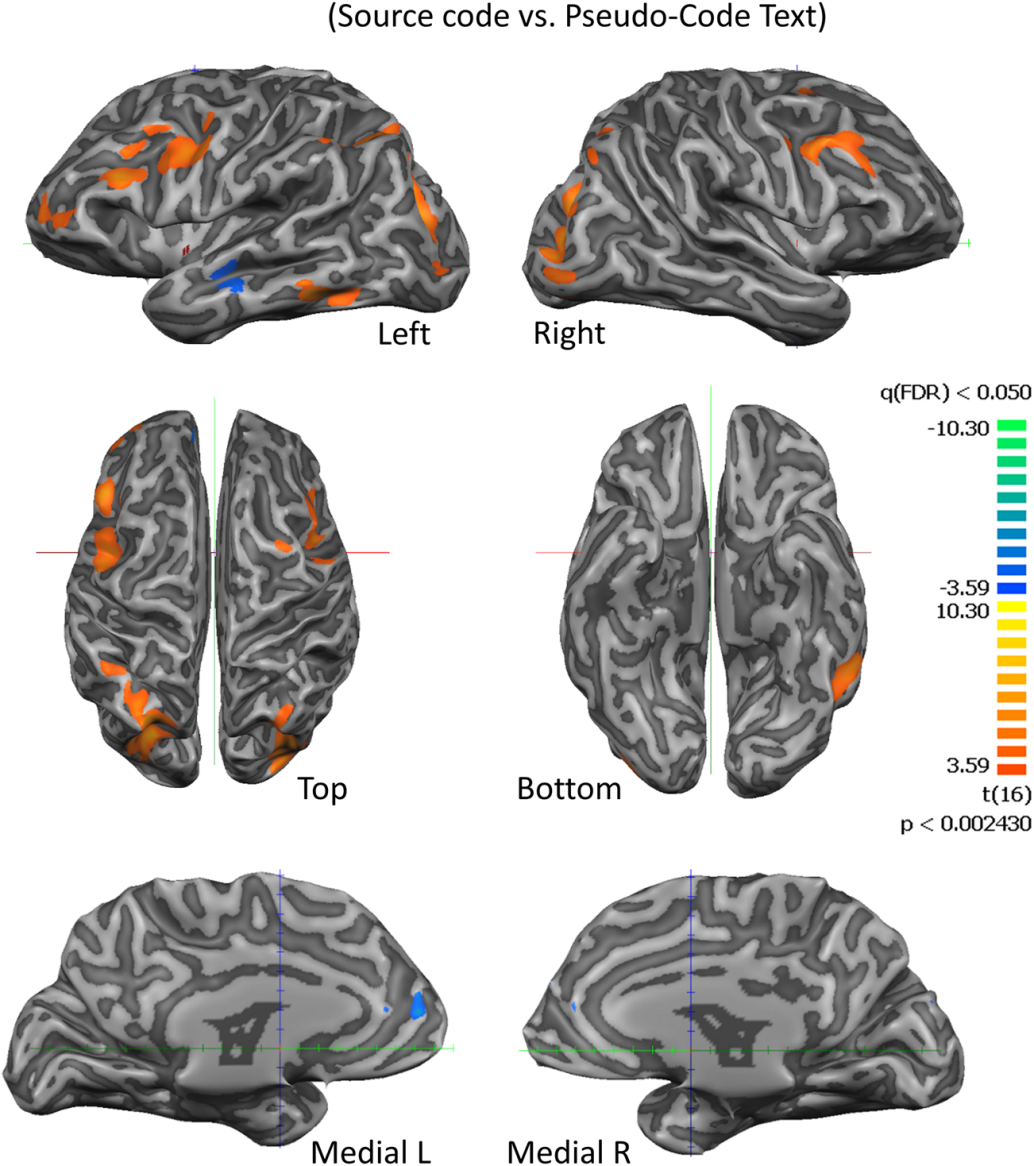
The neural correlates of source code reading and comprehension (source code vs. pseudo-code text). Note that a frontoparietal network is recruited. See Table 1 for regions labels and statistical details

We found a middle frontal, parietal and occipito temporal network that is activated more during software source code understanding than pseudo-code text reading (*t* > 5.49, *p* < 0.00005 (FDR corrected)). Pseudo-code text reading showed increased activation in middle temporal areas (−57, −16, −10; *t* = −4.87, *p* < 0.00017 (FDR corrected)). We found activation in several areas related to program comprehension, language processing (e.g. visual word form area, X = −43, Y = −53, Z = −13 *t* = −4.80, *p* = 0.0001296) (Vigneau et al. 2005), working memory (D’Esposito et al. 1998), error detection and decision-making (BA 9, *p* < 0.00005 (FDR corrected)) (Fig. 2) (Hauke R Heekeren et al. 2008). Table 1 summarizes the significantly activated regions per contrast.

**Table 1:** Regions recruited by source code inspection

	Anatomical Label	Brodmann Area	PeakX	PeakY	PeakZ	T	P-value (FDR p < 0.05)	NrOfVoxels
Understanding Source-code (Source-code vs. Pseudo-Code Text)	R Middle Frontal Gyrus	BA 6	48	5	40	6,73	0,000005	1579
	R Middle Frontal Gyrus	BA 9	51	29	34	5,49	0,000049	342
	R Middle Occipital Gyrus	BA 19	30	−79	10	7,71	0,000001	2069
	L Sup Occipital Gyrus	BA 19	−30	−70	28	8,30	0,000000	4798
	L Precentral Gyrus	BA 6	−42	−1	34	6,21	0,000012	852
	L Middle Frontal Gyrus	BA 9	−48	26	28	6,89	0,000004	579
	L Middle Temporal Gyrus	BA 21	−60	−40	−11	7,21	0,000002	444
	L Mid Temporal Gyrus	BA 21	−57	−16	−11	−4,87	0,000172	702
Searching for a Bug (Source-code with bugs vs. Source-code without bugs)	R Inf Temporal Gyrus	BA 20	57	−46	−11	5,38	0,000062	1333
	L Sup Frontal Gyrus	BA 10	−39	56	25	6,02	0,000018	1252
	L Middle Frontal Gyrus	BA 9	−48	17	31	8,00	0,000001	10,945
	R Middle Occipital Gyrus	BA 18	27	−85	−2	9,40	0,000000	20,927
	L Parietal Lobe, Precuneus	BA 7	−27	−67	40	9,91	0,000000	34,032
	R Middle Frontal Gyrus	BA 8	48	8	40	11,63	0,000000	7743
	L Medial Frontal Gyrus	BA 9	0	47	16	−6,27	0,000011	4297
	R Middle Temporal Gyrus	BA 21	57	−16	−8	−5,94	0,000021	3339
	R Medial Frontal Gyrus	BA 10	15	35	−5	−5,86	0,000024	2713
	L Parietal Lobe, Precuneus	BA 7	0	−37	43	−5,75	0,000030	3434

Summary of the recruited regions for each contrast is reported with the correspondent anatomical localization and statistical values (*P*-values are FDR corrected for multiple comparisons, please note the sign of t values for interpretation of the polarity of significant contrasts). Anatomical label is based on the peak voxel. (R, right; L, left; inf., inferior; mid., middle; med., medial; sup. superior)

**Fig. 3:**
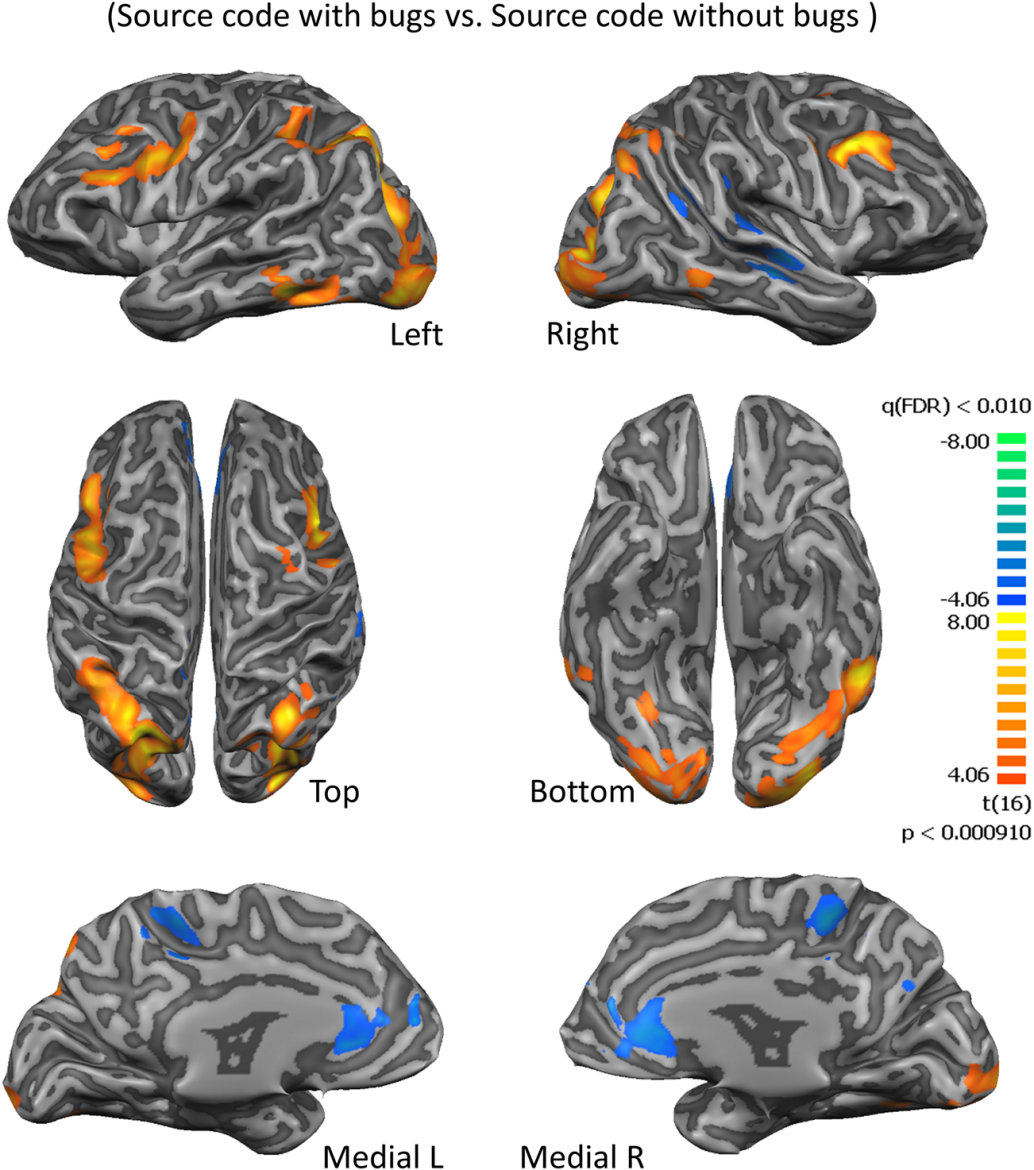
Statistical parametric maps for the ‘searching for a bug’ condition. A network of error monitoring areas and uncertainty related middle frontal areas are activated for the contrast of code with bugs vs. code without bugs

We then compared the ‘searching for a bug’, source code with the bugs with the source code without bugs (neutral). This contrast replicated a similar network of areas (Fig. 3; Table 1) as reported for the above-mentioned contrast. Moreover, ‘search for a bug’ also revealed activation of high-level visual processing and recognition memory regions in the inferior temporal gyrus (BA 20) (Cheung et al. 2009; Desco et al. 2011; Kroger et al. 2008; Tanaka 1992). Other frontal areas related to multiple-task coordination (superior frontal gyrus) (Kennerley et al. 2011; Szameitat et al. 2002, 2006; Wunderlich et al. 2012), error detection and management of uncertainty (middle frontal gyrus) (Eickhoff et al. 2009; Iannaccone et al. 2015) are also activated.

### Decision related regions are activated at the ‘eureka moment’ of bug suspicion in comparison to bug detection

**Fig. 4:**
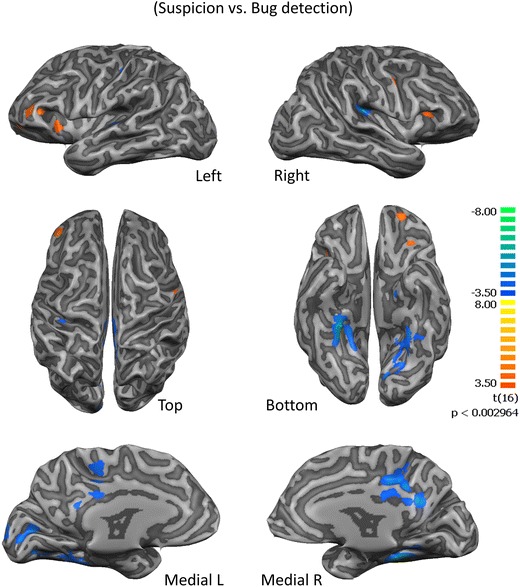
Decision related regions and the insula are activated at the ‘eureka moment’ of bug suspicion in comparison to bug detection. This map represents the areas activated for the statistical contrast of suspicion events with still remaining uncertainty vs. bug detection (certainty or high confidence)

When analyzing the main contrast of interest, the reports of “feeling of suspicion” revealed regions activated for moments of intuition prior to bug detection (the suspicion of a bug, ‘eureka moment’ of uncertainty) in contrast to the bug detection moment (high confidence/certainty). Critically, we found an important role for the insula in relation to this particular contrast (see detailed results in Table 2). Accordingly, the anterior insula is activated by suspicion or intuition of a bug (*t* > 4.11, *p* < 0.0008) as well as small regions in dorso-lateral pre-frontal cortex (Fig. 4). Notably, also an area related to semantic processing in the inferior frontal gyrus (Binder et al. 2009) was also activated by the contrast suspicion vs. bug detection (*t* = 3.46, *p* = 0.0032). On the other hand, the largest region activated during bug confirmation in comparison to bug suspicion was in posterior cingulate (*t* = −6.19, *p* = 0.000013) a region known as a central node of the default mode network (DMN) (Leech and Sharp 2014).

**Table 2:** Regions recruited by ‘Eureka moments’ of bug detection

	Anatomical Label	Brodmann Area	PeakX	PeakY	PeakZ	T	P-value (FDR p < 0.05)	NrOfVoxels
‘Eureka Moments‘(Suspicion vs. Bug confirmation)	L Inf Frontal Gyrus	BA 44	−51	8	19	3,46	0,003241	218
	L Sup Frontal Gyrus	BA 8	−3	23	52	3,73	0,001835	524
	L Precentral Gyrus	BA 6	−39	−7	43	3,77	0,001687	233
	R Anterior Insula	BA 13	30	20	16	4,11	0,000821	939
	R Anterior Cingulate	BA 10	3	53	−2	4,17	0,000724	154
	R Middle Frontal Gyrus	BA 9	51	36	34	4,68	0,000251	443
	R Sup Frontal Gyrus	BA 6	15	26	59	4,68	0,000249	993
	L Middle Frontal Gyrus	BA 47	−30	35	−8	5,08	0,000112	2460
	L Sup Frontal Gyrus	BA 10	−21	50	4	5,37	0,000063	579
	L Middle Frontal Gyrus	BA 46	−42	38	16	5,46	0,000052	2823
	R Posterior Insula	BA 13	36	−28	19	−6,36	0,000009	5202
	R Posterior Cingulate	BA 23	9	−43	25	−6,19	0,000013	17,618
	L Parahippocampal Gyrus	BA 34	−15	−10	−23	−5,01	0,000127	535
	L Postcentral Gyrus	BA 2	−36	−28	40	−4,39	0,000455	875
	R Thalamus		21	−16	19	−4,33	0,000517	627
	R Postcentral Gyrus	BA 43	51	−13	13	−4,16	0,000732	758
	R Parahippocampal Gyrus	BA 28	21	−13	−14	−3,98	0,001066	893
	R Culmen		9	−40	−5	−3,75	0,001737	196
	R Superior Parietal Lobule	BA 7	24	−46	62	−3,67	0,002079	150

A summary of the recruited regions is reported with the correspondent anatomical localization and statistical values (please note the sign of t values for interpretation of the polarity of significant contrasts). Anatomical label is based on the peak voxel. (R, right; L, left; inf., inferior; mid., middle; med., medial; sup. superior)

The strongest activations for the Suspicion vs. Bug detection events (Fig. 4) were found in parietal regions and in the right anterior insula. A less specific contrast of bug detection vs. baseline also reveals a right posterior region (X = 53, Y = −41, Z = 25; Inferior parietal lobule, BA40; *p* < 0.000008) involved in math processing (Desco et al. 2011). On the other hand, the activation of right posterior insula is higher for bug detection than for bug suspicion maybe due to sensorimotor processing (Chang et al. 2013; Rebola et al. 2012) related to the preparation of bug confirmation.

### Activation in the anterior insula correlated with bug detection precision

**Fig. 5:**
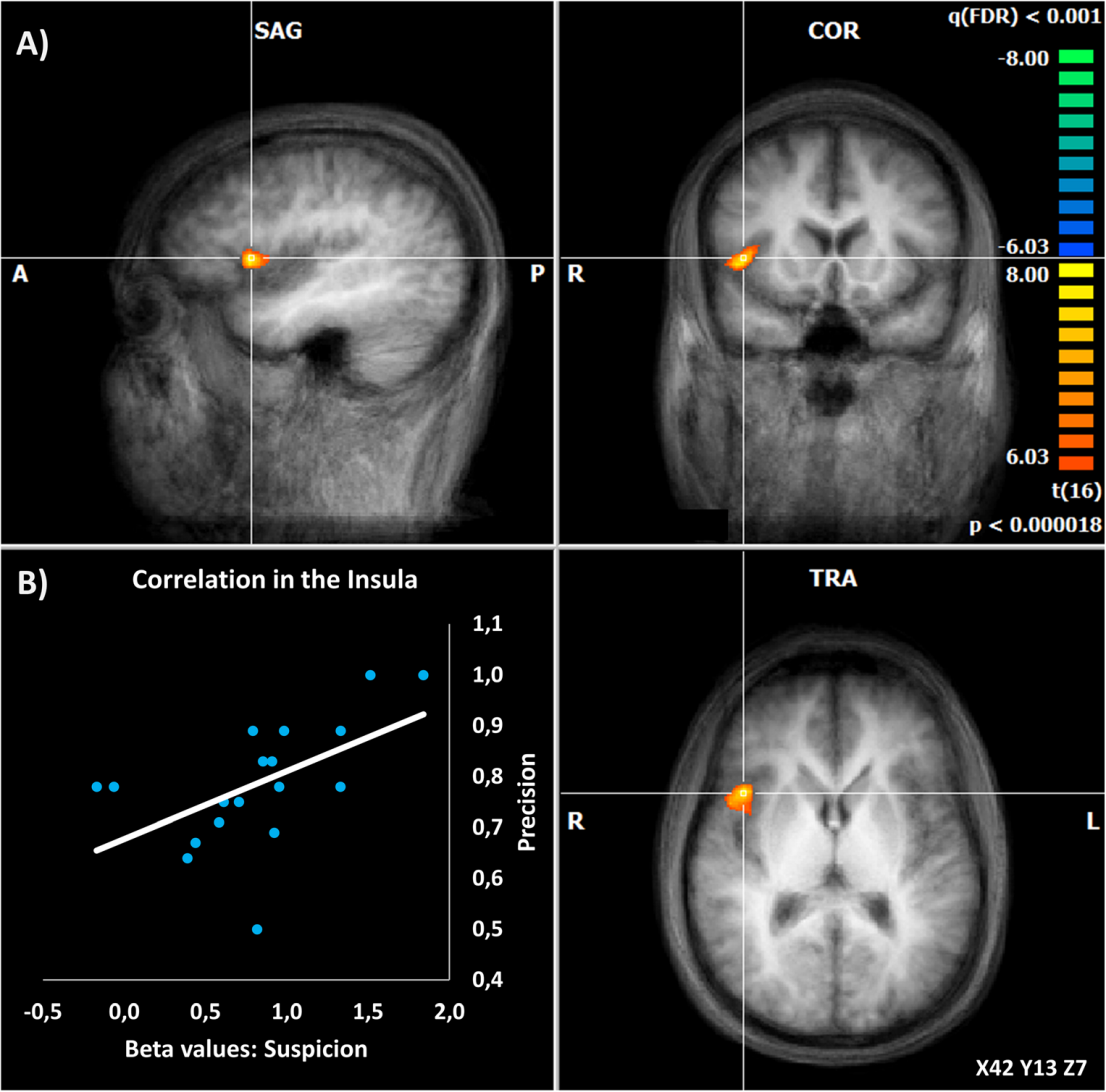
The Insula activates at the very first moment of suspicion and its BOLD signal is correlated with behavioral precision. **a** The activation map shows the insula as activated for the contrast Suspicion vs. baseline. **b** A significant correlation is found between beta values of this contrast in the insula and the individual precision for bug detection by all participants that have precisions over 50% (*r* = 0.540; *p* = 0.021; *N* = 18)

We found a brain region that shows increased activation during the ‘suspicion’ events vs baseline (in the sense of an intuitive feeling of the presence of a bug). This region is located at the right dorsal anterior insula (X = 42, Y = 13, Z = 7; *t* = 8.41; *p* < 0.000018). This contrast shows that this region is highly activated at the very first moment a bug is suspected to be present (Fig. 5a).

The beta values of bug suspicion activation observed in insula under the contrast between bug suspicion and bug detection, were then analyzed in terms of correlation analysis with behavioral precision values. We found that the activity in this insular region at the suspicion event is positively correlated with the precision performance of our group of experts (the % of correct bug detections; *r* = 0.540; *p* = 0.021; Fig. 5b) suggesting that it encodes the quality of the evidence. Notably no correlations were found between activity in other areas (e.g. posterior cingulate; *r* = 0.223, *p* = 0.359) and precision, or the eye tracking fixation parameters of the bug AOI.

### Granger causality analysis reveals a top down modulation network during bug detection

**Fig. 6:**
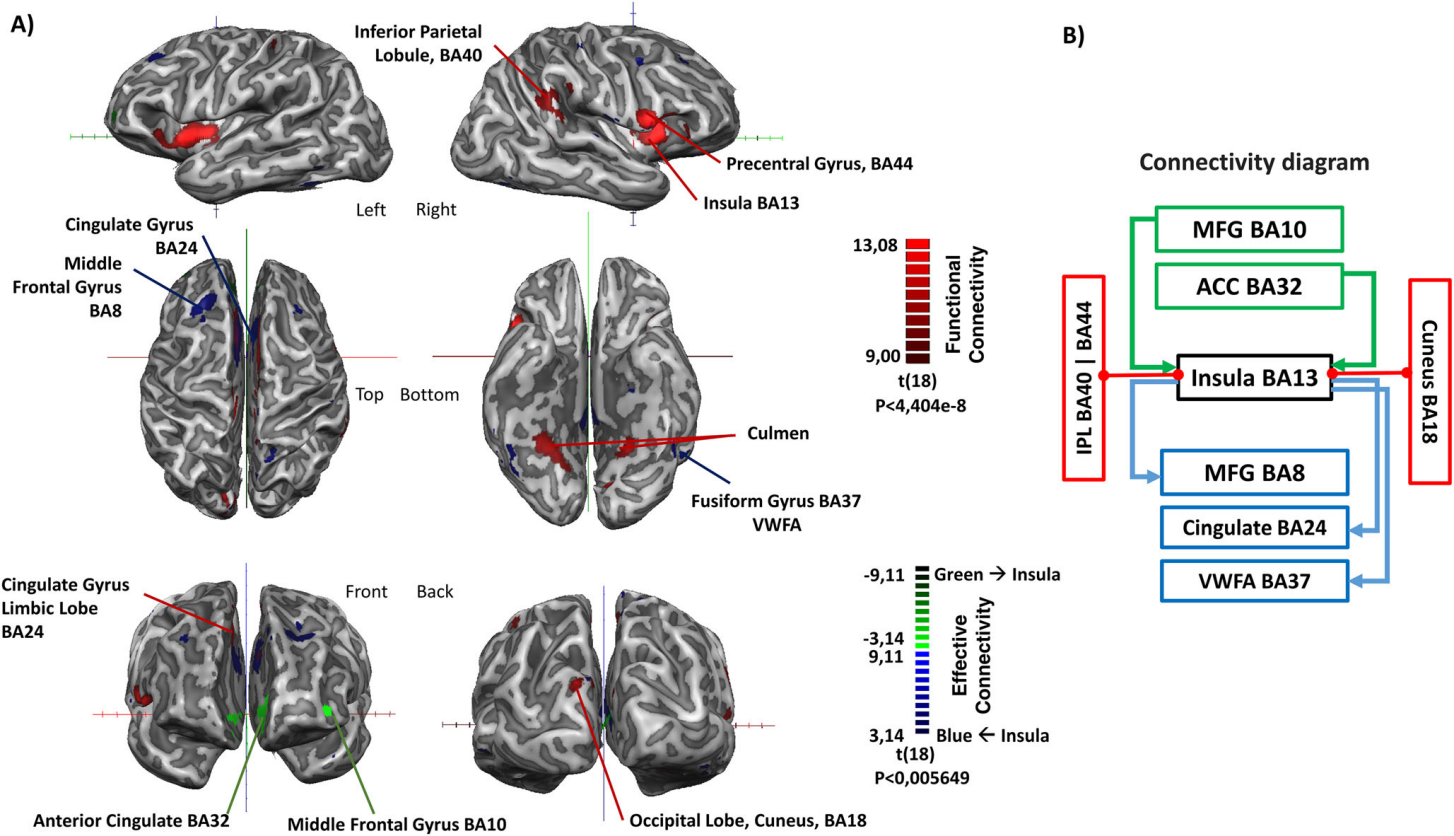
Connectivity analysis reveals a top down influence from the salience network. The cluster in the anterior insula was the seed region for the connectivity analysis of all runs pooled together. A) The Red colormap represents the brain regions showing significant instantaneous connectivity with the insula. The Blue colormap illustrates the areas that are influenced by the insula. In green are projected the areas, which activity significantly influence the insula activation. These areas are labeled in the Fig. B) GCM analysis summary in a form of directional diagram. In sum, the insula receives directed input from the green regions (mainly frontal: anterior cingulate BA32 and middle frontal gyrus BA10) and gives directed input to the blue areas (in particular frontal regions cingulate gyrus BA24 and middle frontal gyrus BA8). This functional integration also includes a path to other sensory (BA18) or math processing regions (BA40). Only clusters surviving the statistical t-test of group average (*p* < 0.007) are reported

Connectivity analysis allowed to investigate the main network of instantaneous correlations and directed influences related to this insular region. Granger causality analysis provided evidence for top-down circuit “reutilization” (from the salience network to parietal regions) for this relatively recent human activity. Anterior areas at the cingulate (BA32, salience network) and middle frontal gyrus (BA10) causally modulate the insula (also belonging to this network) while BA8 in the middle frontal gyrus, cingulate BA24 and fusiform gyrus BA37 receive influence from the insula (Fig. 6; *p* < 0.0057). Most importantly, the latter shows functional influences to earlier math processing parietal regions (BA 40) and higher-level sensory processing regions (BA 18).

## Discussion

Considerable effort has been made to elucidate the neural correlates of decision-making and error monitoring (Iannaccone et al. 2015) across several information processing domains, but not in which concerns evolutionarily recent complex tasks requiring integration of mathematical thinking, abstract language and symbol manipulation such as in software code processing. Many studies stemming from low-level decision tasks (Castelo-Branco and Castelhano 2015), related to visual object classification (or perceptual decision-making (H R Heekeren et al. 2004)) to simple behavioural decisions, suggest an important role of the insula in monitoring conflict and error during decision (Chang et al. 2013; Droutman et al. 2015). The study of such decisions is important but more complex decision-making processes as instantiated by the phylogenetically more recent software-programming framework, and related Eureka decision moments, remain to be understood. We studied decision-making processes and error monitoring while subjects (programming experts in code inspection) performed a bug monitoring and programming (source code related) decision task.

Programming is a relatively new cultural acquisition and it is likely that the brain needs to “reutilize” brain networks for this type of complex activity, by reorganizing this form of complex integrative processing in a top-down manner. In other words, brain regions that are known to be active in response to a specific activity (simple maths) may be alternatively used for other more complex processing domains, when the need arises (Dehaene et al. 2015). This is the case when general language, maths and abstract symbol processing modules need to be integrated. A previous proceedings study (focusing only on the detection of simple syntax errors) considered that language/reading areas were central in understanding these very simple violations (Siegmund et al. 2014) but the task used in that conference report did not require deep integration of language and mathematical thinking during coding inspection. Here, we discovered a set of brain regions with a distinct top-down connectivity pattern which are involved in the complex task studied here, requiring the integration of language/semantic processes (in middle temporal regions), math (middle frontal and inferior parietal areas) and error monitoring/uncertainty and decision-related processes (ACC, inferior and middle frontal gyrus). These regions are functionally connected in deep source code comprehension while expert software programmers are attempting to identify bugs in source code. We found a distinct role for the dorsal insula in deep source code analysis involving integration semantic and mathematical processing. Insula activity levels were critically related to the quality of error detection, involving intuition (bug suspicion) prior to final decision of bug detection, while the ACC causally modulated insular processing within the salience network. A critical novelty in our study is that we included ‘real’ bugs with correct syntax. Given that female programmers still represent a minority (often <15%), our sampling reflected this fact. We had only one female programmer and one limitation of our results is that they cannot be generalized to female programmers.

Most importantly, we identified the neural signatures of the moment when the code reviewer identifies a bug in the code (‘eureka moment’/ suspicion event). Furthermore, these differences in brain activity between suspicion and bug confirmation events are not due to differences in fixation patterns because these did not differ between conditions.

Previous studies reported language related left inferior frontal gyrus and clusters in BA 44, 45 and 46 (frontal areas), superior frontal cortex and right middle temporal gyrus activation related to processing of anomalous sentences (S. D. Newman et al. 2012), and simple syntactic and semantic violations respectively (Kuperberg et al. 2000; A. J. Newman et al. 2001). We show that the network related to the process of ‘searching for a Bug’ also involves other regions, which is to be expected given the integrative nature of our task, and includes areas in inferior parietal cortex, middle frontal gyrus and frontal conflict/error monitoring regions such as the ACC. In fact, these areas subserve high level control functions such as error monitoring, which are important for tasks requiring the use of mathematical skills (Cragg and Gilmore 2014).

Our paradigm is novel because it forces integration of multiple processing domains for complex code analysis. The need for integration across domains is supported by evidence that language and math both require recursive thinking (Maruyama et al. 2012; Semenza et al. 2006), which represents an evolutionary landmark per se. Here, we found evidence for the notion that distributed resources might be used for a more demanding task as source code debugging. Nevertheless, further work is needed to clarify if recursivity in source code may recruit the same regions as language recursive thinking.

Posterior parietal regions were identified to be activated at the moment of Bug detection. These regions have been related to cognitive tasks involving symbolic and non-symbolic numerical form and math processing (Fulbright et al. 2000; Zago et al. 2001). On the other hand, frontal regions related to the salience network including ACC and prefrontal cortex were also activated while performing the bug detection task. These decision and error monitoring (Iannaccone et al. 2015) related brain regions (Botvinick et al. 2004; Krawczyk 2002) were consistently activated during continuously checking if there are bugs in the source code.

We provided evidence that the ACC region causally influences the dorsal anterior insula (also within the salience network (Uddin 2015)) in relation to suspicion. Notably, we did not find activation differences between suspicions about true or false bugs, which is in line with the intuitive nature of bug suspicion (the “feeling” of a bug). At that eureka moment of suspicion the subject is really confident that he/she possibly found a bug, even if later on he/she finds that it is not the case.

It is known that the ability to monitor our own errors is mediated by a network that includes, in addition to the ACC, the dorsomedial prefrontal cortex (dmPFC) and anterior insula (Bastin et al. 2017; Droutman et al. 2015). These authors obtained direct electrophysiological evidence that the anterior insula rapidly detects and conveys error signals to dmPFC. Additionally we show that the very first moment of bug detection (suspicion) activates more the dorsal anterior insula than the bug detection itself. Accordingly, we found that this part of the insula is a region critically involved in the processing of error uncertainty during the emergence of bug suspicion and intuition in a manner that is predictive of future precision. Thus this is in line with the previous reports regarding insula role on tracking arousal variance or error awareness as part of the salience network by integrating information from disparate functional systems involved in general cognition (Chang et al. 2013; Droutman et al. 2015). In this line, our results extend the notion of the insula as region involved in simple perceptual decision-making (Castelhano et al. 2014; Rebola et al. 2012) and suggests that it processes the quality of the evidence. On the other hand, the activation of right posterior insula is higher for bug detection than for bug suspicion which is maybe due to sensorimotor processing (Chang et al. 2013; Rebola et al. 2012) that may occur during detection. Additionally, posterior cingulate is also activated during bug detection in comparison to bug suspicion. This is a region known as a central node of the default mode network (DMN) (Leech and Sharp 2014) and thus this might be reflecting a self-reflection mechanism related to the detection itself.

Furthermore, our work can also be put in the context with the notion that the insula is a key structure within the neural networks involved in decision-making under uncertainty and helps refocusing attention and executive function for better outcome choice (Droutman et al. 2015; Krawczyk 2002). This was further confirmed by Granger causality analysis that demonstrated that in this complex task another region in the salience network, the ACC, causally influences the insula, which in turn influences lower level math related regions. In sum, connectivity analysis revealed a relationship between brain areas for source code related decision making and provided evidence for a network of top-down modulation for bug detection: cingulate (BA32) and anterolateral (BA10) frontal regions causally modulated decision processes in the insula which in turn influenced activity in math and language processing regions. This suggests a network architecture that allows for bug detection in the brain. The instantaneous correlations of these processes show that functional integration (functional connectivity) of insula and math processing regions (possibly related to the first insight of the algorithm in the source code), may indeed be distinct from directed interactions (effective connectivity). These extend to a large set of areas (e.g. underlying decision and executive functions) that explain observed dependencies as also suggested by previous reports (see (Duarte et al. 2017; Karl J Friston 2011)).

By showing a correlation between activity in a specific brain region during error intuition, the insula, and behavioural performance of the participants, we raise the possibility that this brain region signals the quality of programmers’ intuitive capacity to identify bugs when facing the inspection or analysis of challenging code.

In sum, we found that an evolutionary recent and complex task requiring integration across multiple functional domains, elicits a functional connectivity pattern of top down control from the ACC to the insula, a region that is predictive of bug detection accuracy, in the salience network and then to lower level parietal math processing networks. Such a functional organization of information processing during computer program understanding and error checking underlies integration of information from multiple functional modules (maths and logical symbol processing, language) for software source code debugging. Future studies should further elucidate whether cognitive processing of recursive programs do share the same brain circuits as recursive language.

## Electronic supplementary material

ESM 1 (DOC 3926 kb)

ESM 2 (MP4 4916 kb)
