# Supplementary material for: The role of the insula in intuitive expert bug detection in computer code: an fMRI study
Source: Brain Imaging Behav. 2018 May 9;13(3):623–37. doi: 10.1007/s11682-018-9885-1 (PMC6538820; doi:10.1007/s11682-018-9885-1)
Supplement: Supplementary file 1 — (DOC 3926 kb) [file 11682_2018_9885_MOESM1_ESM.doc]

Title - **The role of the insula in intuitive expert bug detection in computer code: an fMRI study.**

**Appendice A**

**Programs used**

Concerning language, dimension and complexity, the following guidelines were used to select the programs where faults were seeded: language, dimension and complexity.

**Language**. We opted for the C programming language. We specifically wanted a mainstream programming language without entering the higher abstraction level of the oriented programming languages. C fulfills that role.

**Dimension**. In order to have several locations to inject faults that are syntactically correct faults (meaning that the compiler would not detect them) and nontrivial (meaning not too obvious), programs must not be too small. At the same time, they should not be so large that the subjects would not able to identify any fault in the allotted time. Starting the several program candidates in preliminary experiments, we concluded that source-code size between 50 and 100 lines would fit our purposes.

**Complexity**. In a similar manner to program size, the algorithms of the programs must not be too simple because any fault seeded would be evident. On the other hand, the algorithms must not be so complex that the subjects would not be able to identify faults with static code analysis alone. Starting with a large set of programs drawn from academic examples and programming contests, we selected the following: matrix multiplication, quicksort and shellsort, which are described next.

**Matrix multiplication** is a simple program were two 2‑dimensional arrays are multiplied (vector multiplication), resulting in a third 2‑dimensional array. This is the simplest and shortest of the three programs where faults were seeded, and it consists in nested loops. **Quicksort** is a recursive algorithm that sorts data by dividing the data set in two and then proceeding to sort each subset until the new subsets become trivial. It is a classic example of recursion. **Shellsort** is a data sorting algorithm. Contrary to quicksort, the shellsort algorithm is not recursive and it is not as easy to follow. The implementation of this algorithm resulted in the more complex of the three programs with faults.

We selected the neutral (calibration) programs only after we have decided upon the programs with faults. This helped us to define the size and complexity for these programs. The neutral programs are String Inversion, Order verification, and Inclusion Verification. **String inversion** is a simple program that reverses the order of the characters in a string. The implementation of this program is recursive. **Order Verification** is a program that verifies if all elements elem_j_ within an integer array verify the following condition: e_i_ < ej < e_k_, for all i<j and k>j. The algorithm of the program is the most complex of the three programs without bugs. **Inclusion Verification** is a program that verifies if a string is contained inside a second string.

**Source-code examples**


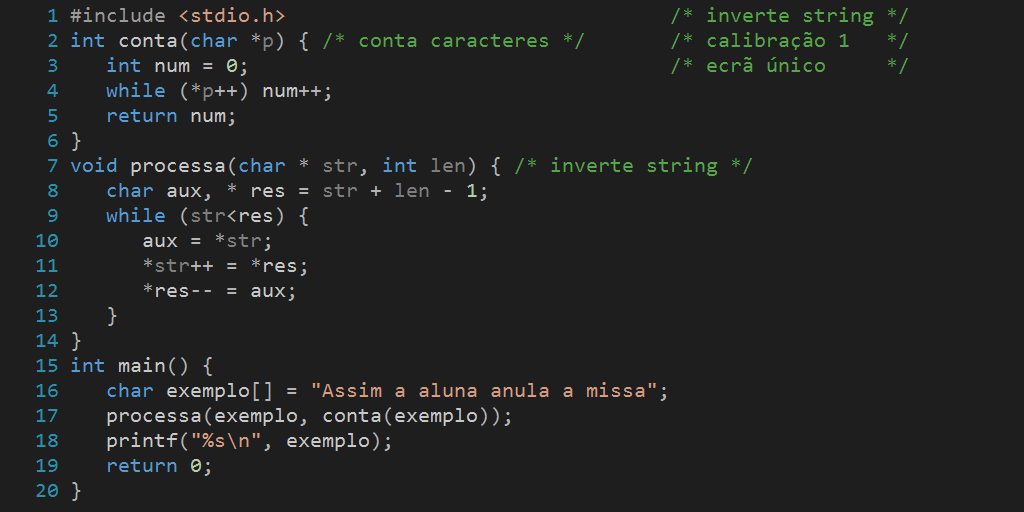


**Fig. A.1** Example of source-code without bugs (neutral code).


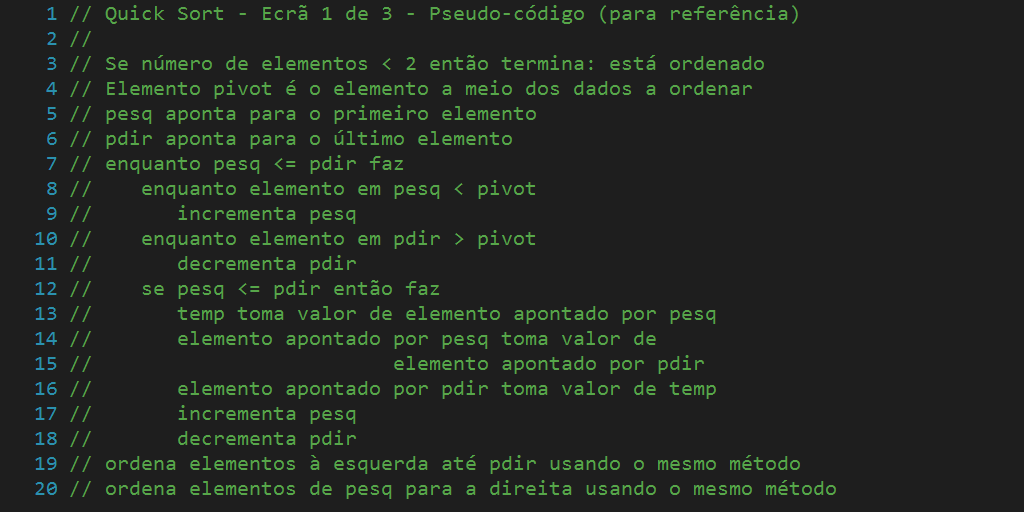


**Fig. A.2** Example of Pseudo-code. A text description of the source-code with bugs. The participants were free to navigate to this screen at any time. The time the participants were spending in these pseudo-code screens were used as predictors of ‘Pseudo-code text’, a reading condition.


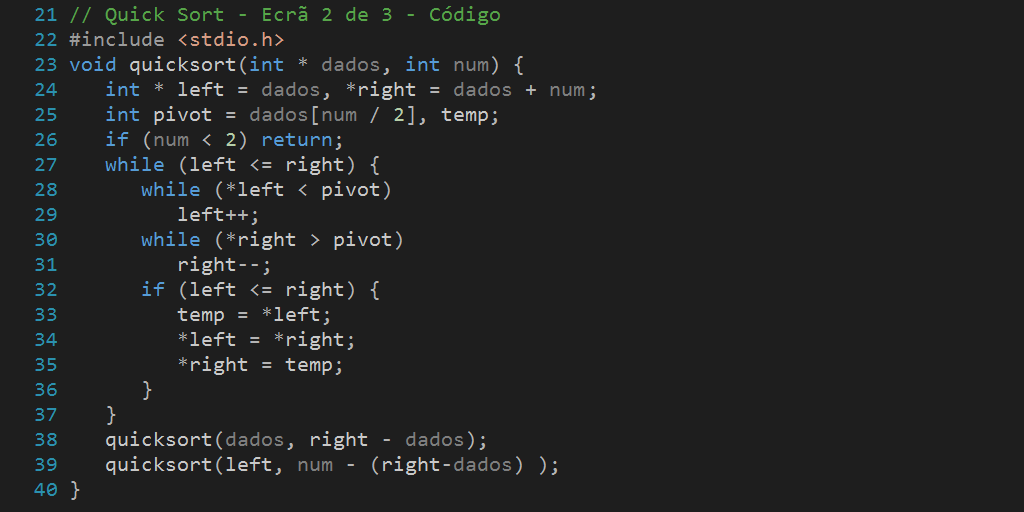


**Fig. A.3**. Example of source code with bugs. This is part of the Quick Sort code used in the experiment. Bugs were present in lines 24 (* right = dados+num **‐1**), 35 (missing **left++;**), 38 (quicksort(dados, right ‐ dados **+1**)) and 39 (quicksort(left, **num ‐ (left‐dados)** )).

**Table A.1**: Detailed number of blocks and durations per condition.

|  | **# Blocks** | | | **Duration [sec]** | | |
| --- | --- | --- | --- | --- | --- | --- |
|  | Avg | Min | Max | Avg | Min | Max |
| **Fixation cross (Baseline)** | 8 | 8 | 8 | 30 | 30 | 30 |
| **Neutral code (No Bugs)** | 3 | 3 | 3 | 123 | 53 | 240 |
| **Bug detection** | 11 | 5 | 18 | 3 | 3 | 3 |
| **Suspicion** | 15 | 7 | 24 | 3 | 3 | 3 |
| **PseudoCode/text** | 12 | 1 | 20 | 32 | 3 | 156 |
| **QuickSort SourceCode Page1** | 5 | 2 | 13 | 109 | 24 | 220 |
| **QuickSort SourceCode Page2** | 2 | 1 | 6 | 65 | 17 | 125 |
| **ShellSort SourceCode Page1** | 8 | 1 | 24 | 81 | 18 | 312 |
| **ShellSort SourceCode Page2** | 3 | 1 | 9 | 62 | 10 | 240 |
| **MatrixMult SourceCode** | 3 | 1 | 8 | 226 | 33 | 590 |
| **Delay Bugs-Suspicion** | n.a. | n.a. | n.a. | 10 | 3 | 52 |

**Table A.2** Summary of Eyetracking results. Data are reported for an area-of-interest analysis (AOI) containing the lines with bugs. The number of fixations and fixation durations are reported for the source code understanding (Bug AOI and Not Bug AOI), suspicion and bug detection events.

|  |  | **Bug AOI** | **Not Bug AOI** | **Suspicion Events** | **Bug Detection Events** |
| --- | --- | --- | --- | --- | --- |
| **# Fixations** | **Median** | 172 | 1001 | 14 | 12 |
|  | **Min** | 8 | 86 | 2 | 2 |
|  | **Max** | 365 | 3798 | 109 | 57 |
| **Fixation Duration [ms]** | **Median** | 291 | 281 | 345 | 408 |
|  | **Min** | 56 | 74 | 84 | 125 |
|  | **Max** | 388 | 379 | 1588 | 1182 |


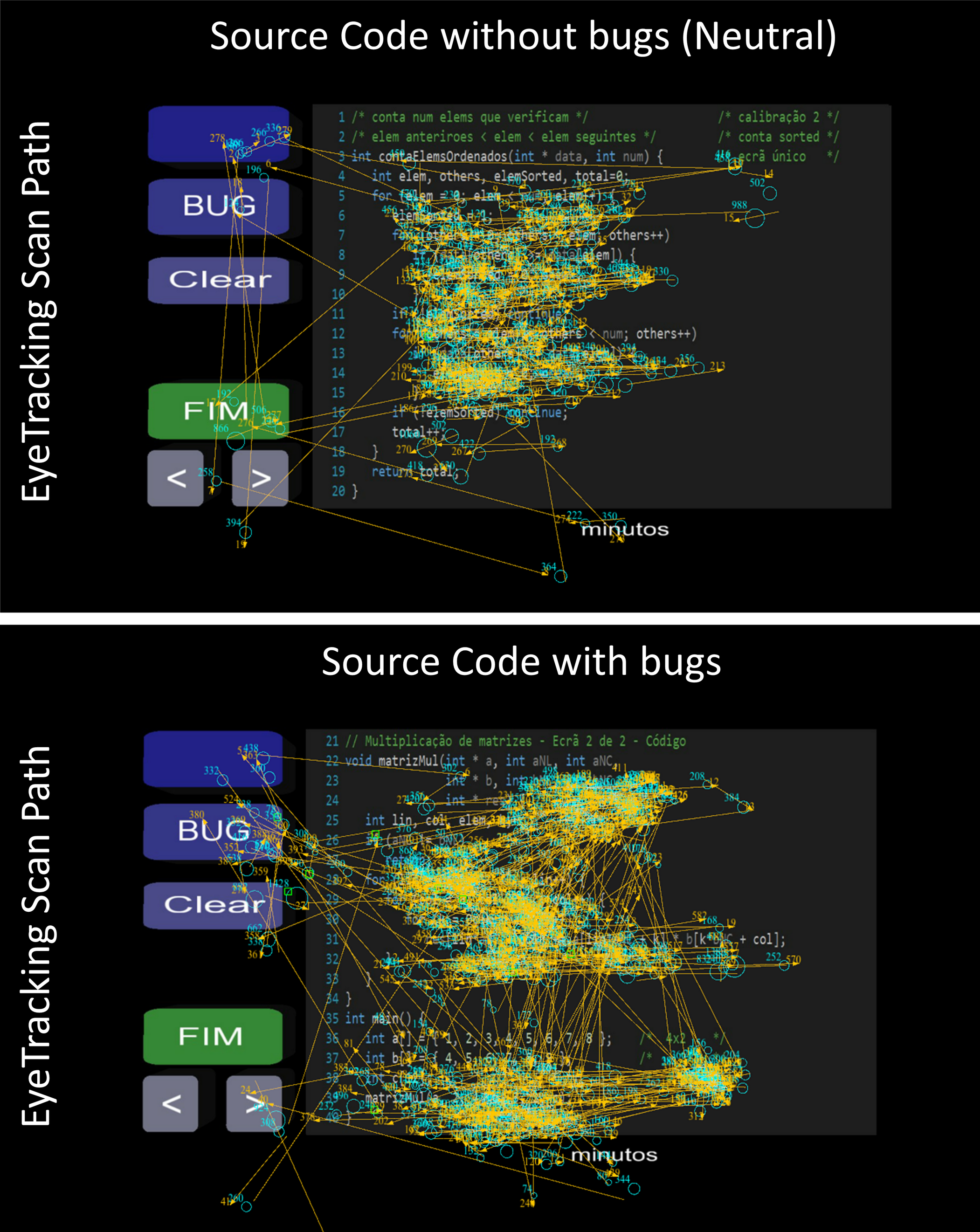


**Fig. A.4** Example sample spatial overlay of eyetracking scanpaths for a trial view of source code debugging from one participant. Blue circles and yellow lines depict fixations and saccades respectively.
